# Association of low muscle mass with cognitive function and mortality in USA seniors: results from NHANES 1999–2002

**DOI:** 10.1186/s12877-024-05035-9

**Published:** 2024-05-11

**Authors:** Yinghui Wang, Dongmei Mu, Yuehui Wang

**Affiliations:** 1 Department of Geriatrics, Jilin Geriatrics Clinical Research Center, The First Hospital of Jilin University, Changchun, 130021 China; 2 Division of Clinical Research, The First Hospital of Jilin University, Changchun, 130021 China; 3 School of Public Health, Jilin University, Changchun, 130021 China

**Keywords:** Low muscle mass, Cognitive function, Mortality, Aging

## Abstract

**Background:**

Sarcopenia and cognitive impairment have been linked in prior research, and both are linked to an increased risk of mortality in the general population. Muscle mass is a key factor in the diagnosis of sarcopenia. The relationship between low muscle mass and cognitive function in the aged population, and their combined impact on the risk of death in older adults, is currently unknown. This study aimed to explore the correlation between low muscle mass and cognitive function in the older population, and the relationship between the two and mortality in older people.

**Methods:**

Data were from the National Health and Nutrition Examination Survey 1999–2002. A total of 2540 older adults aged 60 and older with body composition measures were included. Specifically, 17–21 years of follow-up were conducted on every participant. Low muscle mass was defined using the Foundation for the National Institute of Health and the Asian Working Group for Sarcopenia definitions: appendicular lean mass (ALM) (< 19.75 kg for males; <15.02 kg for females); or ALM divided by body mass index (BMI) (ALM: BMI, < 0.789 for males; <0.512 for females); or appendicular skeletal muscle mass index (ASMI) (< 7.0 kg/m^2^ for males; <5.4 kg/m^2^ for females). Cognitive functioning was assessed by the Digit Symbol Substitution Test (DSST). The follow-up period was calculated from the NHANES interview date to the date of death or censoring (December 31, 2019).

**Results:**

We identified 2540 subjects. The mean age was 70.43 years (43.3% male). Age-related declines in DSST scores were observed. People with low muscle mass showed lower DSST scores than people with normal muscle mass across all age groups, especially in the group with low muscle mass characterized by ALM: BMI (60–69 years: *p* < 0.001; 70–79 years: *p* < 0.001; 80 + years: *p* = 0.009). Low muscle mass was significantly associated with lower DSST scores after adjusting for covariates (ALM: 43.56 ± 18.36 vs. 47.56 ± 17.44, *p* < 0.001; ALM: BMI: 39.88 ± 17.51 vs. 47.70 ± 17.51, *p* < 0.001; ASMI: 41.07 ± 17.89 vs. 47.42 ± 17.55, *p* < 0.001). At a mean long-term follow-up of 157.8 months, those with low muscle mass were associated with higher all-cause mortality (ALM: OR 1.460, 95% CI 1.456–1.463; ALM: BMI: OR 1.452, 95% CI 1.448–1.457); ASMI: OR 3.075, 95% CI 3.063–3.088). In the ALM: BMI and ASMI-defined low muscle mass groups, participants with low muscle mass and lower DSST scores were more likely to incur all-cause mortality ( ALM: BMI: OR 0.972, 95% CI 0.972–0.972; ASMI: OR 0.957, 95% CI 0.956–0.957).

**Conclusions:**

Low muscle mass and cognitive function impairment are significantly correlated in the older population. Additionally, low muscle mass and low DSST score, alone or in combination, could be risk factors for mortality in older adults.

**Supplementary Information:**

The online version contains supplementary material available at 10.1186/s12877-024-05035-9.

## Introduction

Sarcopenia is a common, progressive illness of the skeletal muscles that often causes muscle atrophy, limited mobility, and inadequate muscle strength while also having a significant negative impact on quality of life. Sarcopenia is not lethal, but its presence should be emphasized because it frequently causes other major comorbidities and raises the likelihood of several adverse outcomes, such as falls, diminished physical function, susceptibility, and death [1, 2]. Age, dietary intake, inactivity, illness, and other medical conditions are all associated with sarcopenia. Early adulthood is the time when muscular mass and strength peak. After that, they start to gradually decline, with muscle strength declining more quickly after age 75 [1]. Sarcopenia is a pathological condition when there is an imbalance in the anabolic and catabolic pathways for muscle protein, myofatty degeneration [3], pathogenic interactions between adipose tissue and muscle [4], and disruption of mitochondrial integrity [5, 6]. The recently proposed low muscle mass definitions by the Foundation for the National Institutes of Health (FNIH) [7] and The Asian Working Group for Sarcopenia (AWGS) [8] were applied in this study to assist with the classification of individuals at risk for functional decline.

All older persons undergo cognitive decline, most of which is mild cognitive impairment (MCI), which has minimal impact on daily life [9]. Cognitive impairment is a predictor of disability and mortality in older people, evidenced by degradation of memory, attention, and cognitive performance beyond what would be predicted based on age and degree of education [10]. Notably, the Digit Symbol Substitution Test (DSST), a simple cognitive exam, has been linked to an increased risk of death and physical impairment [11, 12].

Both low muscle mass and cognitive impairment are affected by age and both are associated with mortality. However, the relationship between these two entities in older age groups is unknown. We investigated the association between low muscle mass and DSST on mortality risk and the link between low muscle mass, cognitive function, and long-term mortality. Low muscle mass and DSST together, we thought, could more accurately predict death in older adults.

## Methods

### Survey & study cohort

The National Health and Nutrition Examination Survey 1999–2002 (NHANES) is a nationally representative, complicated, and multistage probability survey that is representative of non-institutionalized, community-dwelling adults. This study performed a secondary analysis of data from this specific survey. The Centers for Disease Control and Prevention has conducted this survey since 1971, and its content can be found at https://www.cdc.gov/nchs/nhanes/index.htm (accessed July 2023). The use of de-identified data exempted this study from review by the local institutional review board.

**Fig. 1:**
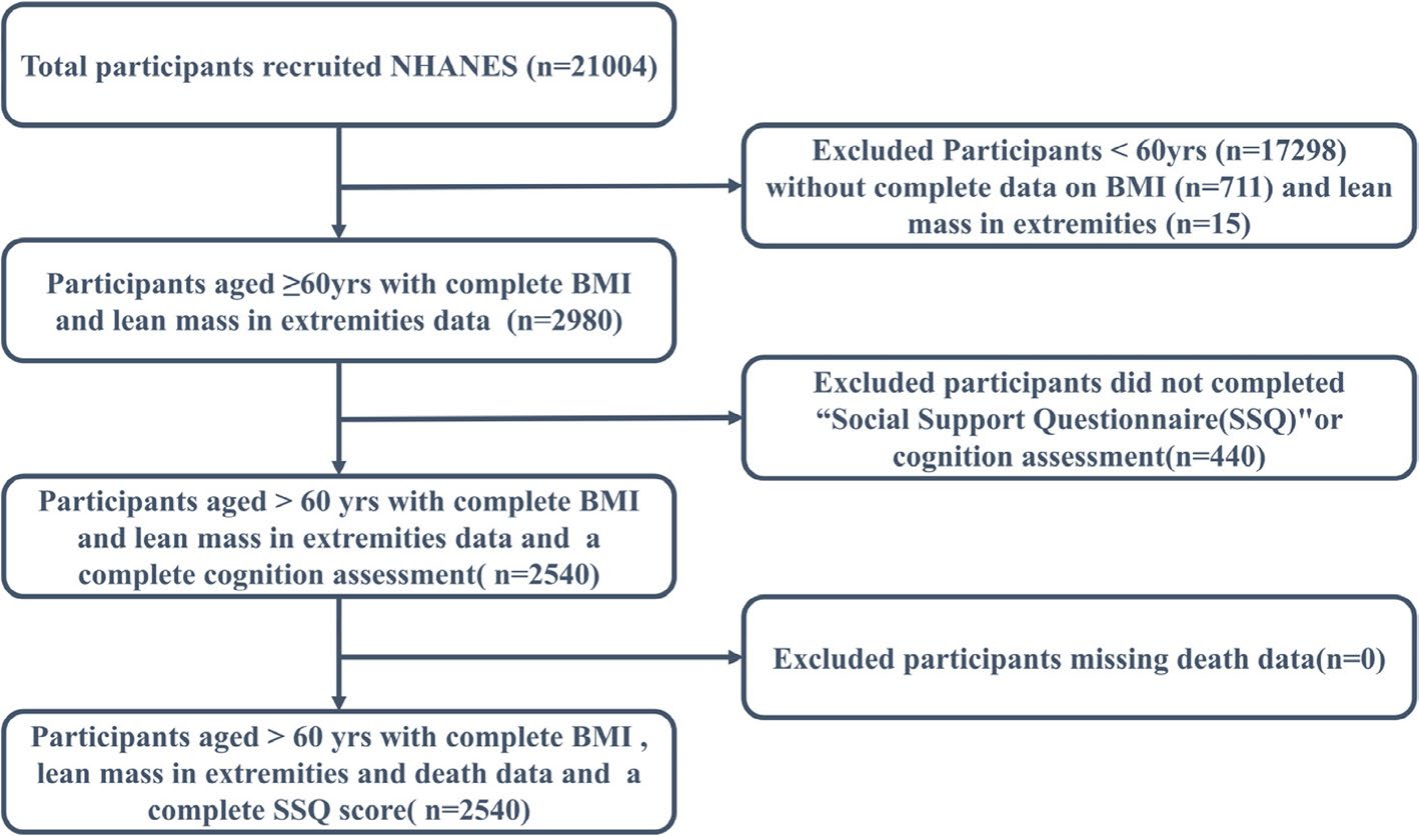
Flow chart of participants in the National Health and Nutrition Examination Survey Study 1999–2002

There were a total of 25,316 participants screened, of which 21,004 were interviewed and 19,759 were examined in a standardized mobile examination center. Our analyses were limited to adults aged 60 years and older (*n* = 3706). After excluding 726 individuals without body composition data and muscle mass data and 440 individuals with missing cognitive assessment data, our final analysis cohort consisted of 2,540 subjects. The flowchart of the study population is visualized in Fig. 1.

### Body composition measures

Body composition (muscle mass and body fat) was evaluated using a QDR-4500 Hologic Scanner (Bedford, MA) for dual energy x-ray absorptiometry (DEXA). The evaluation did not include people who were > 192.5 cm tall or > 136.4 kg heavy. Before the evaluation, all metal was taken out with the exception of hearing aids and fake teeth. The combined fat-free mass for all four limbs (arms and legs) was known as appendicular lean mass (ALM). The appendicular skeletal muscle mass index (ASMI) was defined as the skeletal muscle mass of the extremities divided by the square of height (kg/m^2^).

We use three metrics to define low muscle mass: 1) the FNIH criteria for ALM-defined Low muscle mass (< 19.75 kg in males, < 15.02 kg in females);2) ALM divided by body mass index (BMI) (ALM: BMI, < 0.789 for males; <0.512 for females); and 3) the AWGS criteria for ASMI-defined Low muscle mass (< 7.0 kg/m^2^ for males, < 5.4 kg/m^2^ for females).

Isokinetic strength testing was completed on survey participants aged 50 years and older. A Kin Com MP dynamometer (Chattanooga Group, Inc, Chattanooga, TN) was used to evaluate knee extensor strength, which in turn was used to quantify muscle strength.

### Assessment of cognitive functioning

The only cognitive test used in the NHANES 1999–2002 was the Digit Symbol Substitution Test (DSST), a performance component of the Wechsler Adult Intelligence Scale III. The DSST evaluates working memory, sustained attention, and processing speed [13]. The test is administered using a paper form with a top key that has nine numbers and symbols matched together. The 133 boxes next to the numbers contain corresponding symbols, and participants have 120 s to replicate them. The total number of accurate matches determines the DSST score. A higher score, up to a maximum of 133, indicates stronger cognitive functioning.

### Baseline characteristics

Baseline indicators in this study included demographic characteristics, physical examination and medical comorbidities. All races were included (Non-Hispanic white, Non-Hispanic black, Mexican American, Other Hispanic, Others). Smoking status was categorized as never smoker, ever smoker, and current smoker. Alcohol intake status was categorized by a cut-off value of 12 drinks/year. Physical activity level was categorized according to the level of strenuousness (sitting, walking, light load, and heavy load). The height, weight, waist circumference (WC), and blood pressure (systolic blood pressure and diastolic blood pressure, SBP and DBP) of study participants were tested at a mobile screening center. Body mass index (BMI) was calculated as weight (kilograms) divided by height squared (meters squared). The chronic disease complications in this study included hypertension, diabetes, congestive heart failure (CHF), stroke, cancer, osteoporosis and Arthritis.

### Mortality analysis

Data was obtained through the NHANES 1999–2002 survey which used a probabilistic match to a National Death Index, as well as information from the Social Security Administration to determine mortality status. Mortality data was complete up to December 31, 2019. Cause of death was classified as cardiovascular (including stroke) or other, following the International Statistical Classification of Disease, Injuries and Causes of Death guidelines with the 9th revision used for those dying in 1999, and the 10th revision for all others. Procedures are in place to harmonize the differences in definitions and causes of death. The time of follow-up was calculated in months from the interview date to the date of death or most recent vital record. Vital status was accounted for in > 99% of our sample.

### Statistical analyses

All data were combined into a single data set for analysis. All analyses were weighted using the NHANES analysis program to account for complex stratified sampling. Data are presented as the overall cohort age ≥ 60 years, by the presence/absence of low muscle mass based on the ALM, ALM: BMI, and ASMI definitions. Continuous variables with normal distribution were made using the independent samples t-test, continuous variables with non-normal distribution were made using the Mann-Whitney U rank-sum test, and are expressed as mean (standard deviation, SD). Unsorted count data are expressed as n (%), and comparisons between groups were made using the χ2 test. Multivariable logistic regression models with the calculation of odds ratios (ORs) and 95% confidence intervals (CIs) were used to assess the individual and joint association of low muscle mass and DSST score with all-cause, cardiovascular and cerebrovascular mortality. All multivariable logistic regression models were adjusted for age, gender, race, education, annual household income, smoking status, alcohol intake, hypertension, diabetes mellitus, congestive heart failure, non-skin cancer, stroke, osteoporosis, arthritis, physical activity. Further, Kaplan-Meier plots were used to show survival rates for different low muscle mass defined populations.

All analyses were performed using the statistical packages R version 4.3.1 and SPSS (IBM) version 26, and a two-tailed *P*-value < 0.05 was considered statistically significant.

## Results

### Baseline characteristics stratified by low muscle mass

A total of 2540 US adults aged ≥ 60 and older were included. The prevalence of low muscle mass was 19.0% as defined by ALM, 13.7% as defined by ALM: BMI and 9.2% as defined by ASMI. Basic participant characteristics stratified by low muscle mass are shown in Table 1. Patients with low muscle mass were more likely to be older, better educated, have higher annual household incomes, have stroke, higher waist circumference, systolic blood pressure, serum concentration of total cholesterol, Creatinine, and lower diastolic blood pressure, glucose, and are more likely to be less physically active, and have significantly lower muscular strength than those with normal muscle mass. Population differences in smoking, alcohol use, some laboratory tests (Glycohemoglobin, Triglyceride, low-density lipoprotein, high-density lipoprotein, uric acid, C-reactive protein, fibrinogen), and clinical comorbidities (e.g., Hypertension, Diabetes Mellitus, Congestive Heart Failure, Cancer, Osteoporosis, Arthritis) varied according to the definition of low muscle mass.

**Table 1:** Baseline characteristics of low muscle mass (LMM) in US adults aged 60 years and older in the NHANES 1999–2002

Characteristics	Overall	ALM			ALM: BMI			ASMI		
		LMM (483)	Non-LMM (2057)	*P*-value	LMM (347)	Non-LMM (2193)	*P*-value	LMM (233)	Non-LMM (2307)	*P*-value
Age (years)	70.43 ± 7.33	73.76 ± 7.73	69.66 ± 7.01	< 0.001	73.76 ± 7.73	69.66 ± 7.01	< 0.001	73.76 ± 7.73	69.66 ± 7.01	< 0.001
Gender (%)										
male	1244 (43.3)	120 (17.3)	1124 (49.3)	< 0.001	190 (52.2)	1054 (42.2)	< 0.001	119 (42.6)	1125 (43.3)	< 0.001
female	1296 (56.7)	363 (82.7)	933 (50.7)		157 (47.8)	1139 (57.8)		114 (57.4)	1182 (56.6)	
Race/ethnicity (%)										
Non-Hispanic white	1512 (83.7)	300 (82.5)	1212 (80.4)	< 0.001	187 (83.3)	1325 (83.8)	< 0.001	170 (87.0)	1342 (83.4)	< 0.001
Non-Hispanic black	370 (6.6)	16 (1.6)	354 (7.7)		10 (1.7)	360 (7.2)		9 (1.5)	361 (7.1)	
Mexican American	508 (2.8)	122 (3.7)	386 (2.6)		129 (6.0)	379 (2.4)		36 (2.3)	472 (2.8)	
Other Hispanic	97 (4.4)	26 (6.9)	71 (3.8)		16 (6.4)	81 (4.1)		10 (4.9)	87 (4.3)	
Others	53 (2.5)	19 (5.3)	34 (1.9)		5 (2.6)	48 (2.5)		8 (4.3)	45 (2.4)	
Educational attainment (%)										
Less than high school	1012 (29.1)	208 (31.9)	804 (28.5)	< 0.001	183 (37.8)	829 (28.0)	< 0.001	95 (33.6)	917 (28.6)	< 0.001
High school graduate	611 (29.6)	118 (31.1)	493 (29.2)		79 (30.8)	532 (29.4)		52 (25.6)	559 (30.0)	
More than high school	912 (41.3)	154 (37.0)	758 (42.3)		84 (31.4)	828 (42.6)		86 (40.8)	826 (41.4)	
Annual household income (%)										
<$20,000	740 (30.5)	180 (41.1)	560 (28.2)	< 0.001	129 (37.8)	611 (29.6)	< 0.001	88 (40.3)	652 (29.5)	< 0.001
$20,000-$75,000	1152 (54.4)	179 (48.5)	973 (55.8)		139 (53.7)	1013 (54.5)		90 (47.5)	1062 (55.1)	
>$75,000	280 (15.1)	37 (10.4)	243 (16.0)		22 (8.5)	258 (15.9)		22 (12.2)	158 (15.4)	
Co-Morbid Conditions (%)										
Hypertension	1295 (49.9)	222 (49.4)	1073 (50.0)	< 0.001	175 (50.6)	1120 (49.8)	< 0.001	98 (43.1)	1197 (50.5)	< 0.001
Diabetes Mellitus	421 (13.9)	54 (9.2)	367 (15.0)	< 0.001	65 (15.2)	356 (13.8)	< 0.001	12 (4.2)	409 (14.9)	< 0.001
Congestive Heart Failure	152 (5.8)	27 (5.6)	125 (5.8)	< 0.001	34 (9.4)	118 (5.3)	< 0.001	14 (5.6)	138 (5.8)	< 0.001
Cancer	473 (21.1)	98 (23.3)	375 (20.5)	< 0.001	57 (17.9)	416 (21.5)	< 0.001	59 (26.7)	414 (20.5)	< 0.001
Stroke	155 (5.6)	34 (6.9)	121 (5.3)	< 0.001	33 (8.4)	122 (5.2)	< 0.001	23 (7.2)	132 (5.4)	< 0.001
Osteoporosis	263 (13.2)	102 (25.1)	162 (10.5)	< 0.001	37 (13.6)	227 (13.1)	< 0.001	37 (18.4)	227 (12.7)	< 0.001
Arthritis	1183 (49.2)	208 (48.3)	975 (49.4)	< 0.001	168 (54.1)	1015 (48.6)	< 0.001	89 (41.7)	1094 (49.9)	< 0.001
Smoking status (%)										
Nonsmoker	1187 (47.1)	249 (52.8)	938 (45.8)	< 0.001	160 (47.3)	1027 (47.1)	< 0.001	96 (44.7)	1091 (47.4)	< 0.001
Former smoker	1045 (40.7)	157 (16.1)	888 (11.3)		150 (42.2)	895 (40.5)		89 (20.3)	956 (11.4)	
Current smoker	308 (12.2)	77 (31.1)	231 (42.9)		37 (10.5)	271 (12.4)		48 (35)	260 (41.2)	
Alcohol intake ≥ 12 drinks/year (%)	1495 (60.5)	225 (49.6)	1270 (61.1)	< 0.001	194 (58.5)	1301 (59.0)	< 0.001	123 (51.8)	1372 (59.7)	< 0.001
Moderate/vigorous physical activities (%)	1203 (51.2)	186 (41.7)	1017(53.4)	< 0.001	113 (33.5)	1090 (53.5)	< 0.001	91 (41.1)	1112 (52.3)	< 0.001
Anthropometric Measures										
BMI (kg/m²)	28.18 ± 5.49	23.18 ± 3.41	29.30 ± 25.23	< 0.001	30.81 ± 5.36	27.84 ± 5.42	< 0.001	21.34 ± 2.54	28.87 ± 5.23	< 0.001
WC (cm)	99.90 ± 13.62	101.15 ± 13.89	99.61 ± 13.54	0.346	101.34 ± 13.60	99.72 ± 13.61	0.204	100.32 ± 13.96	99.85 ± 13.58	0.585
SBP (mmHg)	138.62 ± 21.71	142.77 ± 23.56	137.67 ± 21.15	< 0.001	142.77 ± 23.56	137.67 ± 21.15	0.005	142.77 ± 23.56	137.67 ± 21.15	0.804
DBP (mmHg)	69.17 ± 15.58	66.78 ± 14.9	69.71 ± 15.68	< 0.001	66.78 ± 14.9	69.71 ± 15.68	0.156	66.78 ± 14.9	69.71 ± 15.68	< 0.001
DSST score	46.89 ± 17.83	43.67 ± 18.49	47.63 ± 17.59	< 0.001	40.01 ± 17.92	47.77 ± 17.62	< 0.001	41.86 ± 18.39	47.41 ± 17.69	0.001
Muscle measures										
ALM (kg)	20.91 ± 5.70	14.35 ± 2.13	22.42 ± 5.18	< 0.001	18.79 ± 4.87	21.18 ± 5.74	< 0.001	15.53 ± 3.74	21.46 ± 5.58	< 0.001
ALM: BMI	0.75 ± 0.18	0.63 ± 0.12	0.78 ± 0.18	< 0.001	0.61 ± 0.13	0.77 ± 0.18	< 0.001	0.73 ± 0.17	0.75 ± 0.18	0.315
ASMI (kg/m^2^)	7.52 ± 1.46	5.81 ± 0.68	7.91 ± 1.30	< 0.001	7.39 ± 1.34	7.54 ± 1.47	0.021	5.70 ± 0.81	7.70 ± 1.38	< 0.001
Strength-Peak force (Newtons)	324.48 ± 111.46	232.31 ± 62.42	344.45 ± 109.69	< 0.001	301.62 ± 94.10	327.14 ± 113.01	< 0.001	229.51 ± 69.33	333.62 ± 110.48	< 0.001
Laboratory tests										
Glucose (mmol/L)	5.73 ± 1.96	5.38 ± 1.32	5.81 ± 2.06	0.001	5.72 ± 1.78	5.73 ± 1.98	0.025	5.31 ± 0.83	5.78 ± 2.03	0.293
Glycohemoglobin (%)	5.79 ± 1.03	5.56 ± 0.91	5.84 ± 1.05	< 0.001	5.85 ± 0.94	5.78 ± 1.04	0.002	5.42 ± 0.46	5.82 ± 1.06	< 0.001
Total cholesterol (mmol/L)	5.52 ± 1.05	5.62 ± 0.98	5.49 ± 1.07	0.033	5.53 ± 1.12	5.51 ± 1.04	0.241	5.55 ± 1.02	5.51 ± 1.06	0.934
Triglyceride (mmol/L)	1.81 ± 1.01	1.67 ± 0.81	1.84 ± 1.05	0.323	1.89 ± 0.97	1.80 ± 1.02	0.010	1.59 ± 0.91	1.83 ± 1.02	0.016
Low-density lipoprotein (mmol/L)	3.30 ± 0.92	3.17 ± 0.81	3.34 ± 0.94	0.061	3.35 ± 1.01	3.30 ± 0.91	0.366	3.15 ± 0.90	3.32 ± 0.92	0.021
High-density lipoprotein (mmol/L)	1.38 ± 0.43	1.65 ± 0.50	1.32 ± 0.39	< 0.001	1.32 ± 0.38	1.39 ± 0.44	0.005	1.62 ± 0.50	1.36 ± 0.42	< 0.001
Uric acid (µmol/L)	336.97 ± 90.05	301.86 ± 87.02	344.98 ± 8.82	< 0.001	348.50 ± 86.03	335.56 ± 90.43	0.358	310.89 ± 89.67	339.63 ± 89.67	< 0.001
Creatinine (µmol/L)	80.05 ± 44.01	73.26 ± 40.84	81.58 ± 44.55	< 0.001	81.90 ± 44.17	79.82 ± 43.98	0.025	80.72 ± 47.94	79.98 ± 43.59	0.934
C-reactive protein (mg/dL)	0.54 ± 0.93	0.49 ± 0.82	0.55 ± 0.96	0.012	0.73 ± 0.96	0.52 ± 0.93	< 0.001	0.48 ± 0.97	0.55 ± 0.93	< 0.001
Fibrinogen (mg/dL)	380.02 ± 76.4	377.88 ± 75.63	380.57 ± 6.56	0.879	411.54 ± 85.4	376.62 ± 74.57	< 0.001	391.80 ± 3.54	379.04 ± 75.65	0.389

All estimates accounted for sample weights and complex survey designs, and means and percentages were adjusted for survey weights of NHANES

*LMM *Low muscle mass

*ALM* Appendicular lean mass

ALM defined LMM is defined as an appendicular lean mass <19.75kg in men, or <15.02kg in females

ALM:BMI defined LMM is defined as ALM:BMI ratio <0.789 and <0.512

ASMI defined LMM is defined as ASMI <7.0kg/m^2^ and <5.4kg/m^2^

### Association of low muscle mass with cognitive function

DSST scores differed by age and muscle mass (Additional file 1: Table S1). DSST scores decreased significantly with age (ALM: *p* < 0.001; ALM: BMI: *p* < 0.001; ASMI: *p* < 0.001). The rank-sum test was used to compare the differences in DSST scores for different muscle mass statuses across the three age subgroups, and it was discovered that while the means of DSST scores were not all statistically significant across the groups, the DSST scores for non-LMM statuses were higher in almost every age subgroup. The results of the associations of low muscle mass with DSST scores are presented in Table 2. In the fully adjusted models (Model 3), Low muscle mass was strongly associated with DSST scores (ALM: 43.56 ± 18.36 vs. 47.56 ± 17.44, *p* < 0.001; ALM: BMI: 39.88 ± 17.51 vs. 47.70 ± 17.51, *p* < 0.001; ASMI: 41.07 ± 17.89 vs. 47.42 ± 17.55, *p* < 0.001).

**Table 2:** Cross-sectional association between low muscle mass (LMM) and cognitive score

Cognitive functioning (DSST)			
	Model 1	Model 2	Model 3
ALM			
LMM	43.67 ± 18.49	43.67 ± 18.35	43.56 ± 18.36
Non-LMM	47.63 ± 17.59	47.45 ± 17.40	47.56 ± 17.44
*P*-value	< 0.001	< 0.001	< 0.001
ALM: BMI			
LMM	40.01 ± 17.92	39.73 ± 17.36	39.88 ± 17.51
Non-LMM	47.77 ± 17.62	47.64 ± 17.47	47.70 ± 17.51
*P*-value	< 0.001	< 0.001	< 0.001
ASMI			
LMM	41.86 ± 18.39	41.61 ± 18.01	41.07 ± 17.89
Non-LMM	47.41 ± 17.69	47.30 ± 17.51	47.42 ± 17.55
*P*-value	< 0.001	< 0.001	< 0.001

All values are adjusted mean ± SD

Data are weighted according to the National Health and Nutrition Examination Survey protocol

Model 1: no adjustment

Model 2: adjusted for age, gender, race, education, annual household income, smoking status, alcohol intake

Model 3: adjusted for model 2 plus hypertension, diabetes mellitus, congestive heart failure, non-skin cancer, stroke, osteoporosis, arthritis, physical activity

### Association of low muscle mass with mortality

**Fig. 2:**
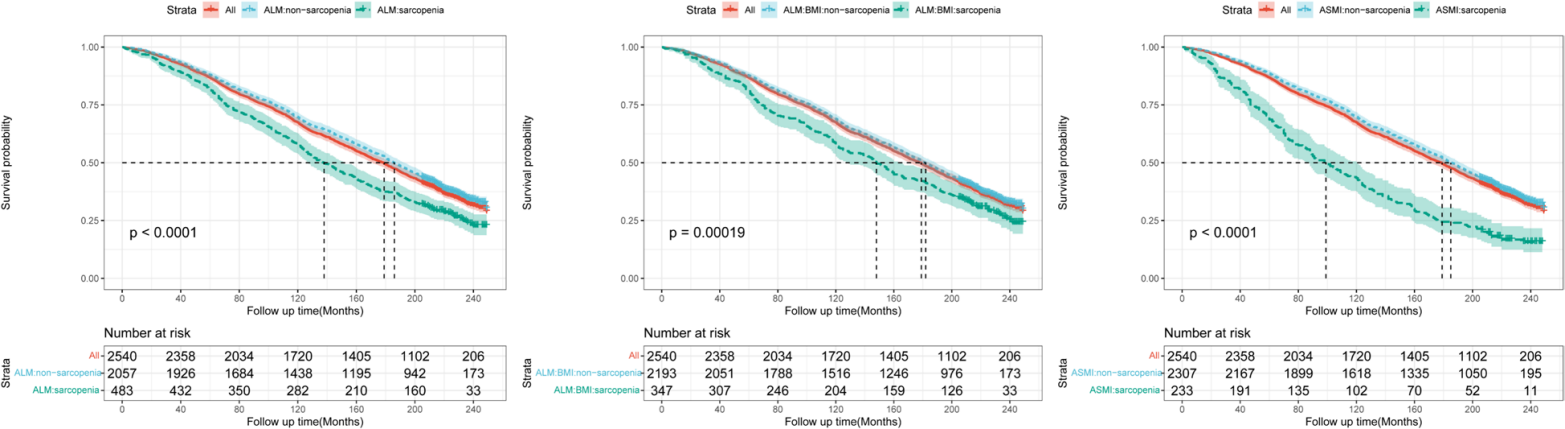
Kaplan-Meier plots of survival rates according to low and non-low muscle mass individuals

By the end of follow-up (median of 157.8 months), 63.6% of enrollees died, of which 27.7% were cardiovascular deaths and 5.6% cerebrovascular deaths. After adjusting for covariates, low muscle mass was strongly associated with all-cause mortality (ALM: OR 1.460, 95% CI 1.456–1.463; ALM: BMI: OR 1.452, 95% CI 1.448–1.457; ASMI: OR 3.075, 95% CI 3.063–3.088). However low muscle mass was not significantly associated with increased risk of cardiovascular mortality or cerebrovascular mortality (Additional file 1: Table S2). Unadjusted Kaplan-Meier estimates of death were obtained according to muscle mass subgroups (Fig. 2). Regardless of the definition of low muscle mass, the overall mortality rate was much higher in those with low muscle mass than in those without low muscle mass (*p* < 0.001).

### Joint association of low muscle mass and cognitive function with mortality

Univariate and multivariate models of low muscle mass and DSST associated with mortality are presented in Table 3. DSST was significantly associated with total mortality regardless of the presence of low muscle mass (LMM/Non-LMM (ALM): OR 0.994, 95% CI 0.994–0.994/OR 0.983, 95% CI 0.983–0.983; LMM/Non-LMM (ALM: BMI): OR 0.972, 95% CI 0.972–0.972/ OR 0.984, 95% CI 0.984–0.984; LMM/Non-LMM (ASMI): OR 0.957, 95% CI 0.956–0.957/ OR 0.986, 95% CI 0.985–0.986). This association was more pronounced in the LMM population defined by ALM: BMI and ASMI. That is, patients with LMM had a stronger link between low DSST and an increased risk of all-cause mortality. In addition, the link between DSST and all-cause mortality did not differ significantly across each of the ALM-defined muscle mass categories. DSST was not significantly associated with cardiovascular death or cerebrovascular death, which may be related to the relatively small number of people who experience cardiovascular or cerebrovascular death.

**Table 3:** Logistic regression modeling on low muscle mass (LMM) and cognitive function and mortality

	ALM		ALM: BMI		ASMI	
	LMM	Non-LMM	LMM	Non-LMM	LMM	Non-LMM
Overall Death						
Model 1	0.970(0.970–0.970)	0.960(0.959–0.960)	0.961(0.961–0.961)	0.962(0.962–0.962)	0.940(0.940–0.941)	0.963(0.963–0.963)
Model 2	0.990(0.990–0.990)	0.980(0.980–0.980)	0.981(0.980–0.981)	0.982(0.982–0.982)	0.973(0.973–0.973)	0.983(0.982–0.983)
Model 3	0.994(0.994–0.994)	0.983(0.983–0.983)	0.972(0.972–0.972)	0.984(0.984–0.984)	0.957(0.956–0.957)	0.986(0.985–0.986)
Cardiovascular Death						
Model 1	1.005(1.005–1.006)	0.999(0.999–0.999)	1.004(1.004–1.004)	1.001(1.001–1.001)	0.988(0.988–0.988)	1.002(1.002–1.002)
Model 2	1.024(1.024–1.024)	1.002(1.002–1.002)	1.022(1.021–1.022)	1.004(1.004–1.005)	0.995(0.995–0.995)	1.008(1.008–1.008)
Model 3	1.037(1.036–1.037)	1.006(1.006–1.006)	1.031(1.030–1.031)	1.009(1.009–1.009)	1.014(1.013–1.014)	1.011(1.011–1.012)
Cerebrovascular Death						
Model 1	0.988(0.988–0.988)	0.971(0.971–0.972)	0.972(0.972–0.972)	0.975(0.975–0.976)	0.975(0.975–0.976)	0.974(0.973–0.974)
Model 2	0.999(0.999–0.999)	0.970(0.970–0.970)	0.965(0.965–0.966)	0.977(0.976–0.977)	0.994(0.993–0.994)	0.973(0.973–0.973)
Model 3	0.997(0.997–0.997)	0.968(0.967–0.968)	0.957(0.956–0.958)	0.977(0.977–0.977)	1.004(1.003–1.005)	0.974(0.973–0.974)

All values represented are hazard ratios [95% confidence interval]

Data are weighted according to the National Health and Nutrition Examination Survey protocol

Model 1: no adjustment

Model 2: adjusted for age, gender, race, education, annual household income, smoking status, alcohol intake

Model 3: adjusted for model 2 plus hypertension, diabetes mellitus, congestive heart failure, non-skin cancer, stroke, osteoporosis, arthritis, physical activity

## Discussion

The preliminary results imply that older persons with low muscle mass, regardless of age, had lower DSST scores. To the best of our knowledge, this is the first study that uses nationally representative data to look at the combined impact of low muscle mass and cognitive function on all-cause death (with 17–21 years of follow-up) among older people over 60 in American communities.

Sarcopenia, a health issue that needs immediate attention in older people and seriously affects the quality of life, is a progressive reduction in skeletal muscle mass and strength that happens with aging [14]. According to the results of our investigation, DSST also gradually decreases with age. The DSST has been demonstrated to be an accurate assessment of processing speed, executive functioning, and working memory impairments and can be used as a reliable measure of functioning. It is a sensitive test for detecting cognitive problems [11, 15]. Higher DSST scores indicate improved cognitive functioning; nevertheless, there is no gold standard for threshold scores on cognitive tests that assess cognitive impairment. The current study’s mean DSST was 46.89 ± 17.83, with the lowest quartile (DSST ≤ 34) indicating poor cognitive performance or impairment (consistent with the methodology used in the previous literature) [16, 17]. The majority of the study population was in mild cognitive decline, with only the ALM: BMI-defined LMM population over the age of 80 years showing significant cognitive dysfunction (DSST: 32.40 ± 14.42). Low muscle mass and cognitive scores had a negative correlation in cross-sectional studies, especially in the age group of 60 to 79 years (ALM: *p* < 0.001; ALM: BMI: *p* < 0.001; ASMI: *p* < 0.001). After controlling for confounders, the relationship between low muscle mass and DSST remained significant. This is in line with what other cross-sectional studies [18, 19] and meta-analyses [20–23] have discovered. Similar results were found in a recent cross-sectional study of Chinese older adults, which suggested that older adults with probable sarcopenia [95% confidence interval (CI): 1.06–1.91, *P* = 0.017] and sarcopenia (95% CI: 1.04–2.85, *P* = 0.035) were more likely to develop new-onset MCI. Individuals with sarcopenia were 1.72 times more likely to develop MCI than those without sarcopenia [19]. ALM, however, was not connected to the development of cognitive impairment in a prospective study that examined the relationship between physical function and cognitive function [24]. A 24-month structured, moderate-intensity muscle-training exercise for older persons did not improve general or domain-specific cognitive function when compared to a health education program for inactive older adults [25]. This discrepancy most likely results from different muscle mass and cognitive impairment screening methods. The NHANES cohort had a higher degree of representativeness and fewer sample mistakes, and this study further divided the participants based on age and different low muscle mass classifications. We conducted a cross-sectional study to assess the relationship between low muscle mass and DSST, and more prospective studies are needed to determine whether low muscle mass increases the risk of cognitive impairment.

Inadequate nutritional intake [26, 27] and decreased physical exercise [28] may be the common factors behind the positive association between sarcopenia and cognitive impairment. Additionally, several chronic metabolic conditions brought on by inadequate nutrition and physical inactivity might exacerbate sarcopenia and cognitive decline [29]. Skeletal muscle secretes myokines and peptides that safeguard brain structure and function, including cognition, through mediating muscle-organ interaction [28, 30, 31]. On the other hand, cognitive decline can cause sarcopenia by lowering physical activity [30, 32]. Muscle strength, muscle mass, and physical performance were linked to cognitive function in a prospective cohort study of middle-aged and older men, and a lower DSST may be linked to a more severe stage of muscle function loss [33]. The DSST, as a multifactorial test, is sensitive enough to represent a fairly diverse range of types (visual perceptual, oculomotor, fine manual motor, and mental functions) of cognitive situations [34]. Even though our study solely looked at the connection between DSST and low muscle mass, its clinical relevance should not be disregarded.

The impact of low muscle mass versus DSST on mortality in older persons was also examined. Both univariate and multivariate analyses showed that all-cause mortality was higher in low muscle mass patients than in older persons with normal muscle mass, which is consistent with other studies. Additionally, low muscle mass was exclusively linked to all-cause mortality in our analysis; it was not linked to cardiovascular or cerebrovascular mortality. DSST was connected to all-cause mortality in the fully adjusted model, regardless of whether the patient had low muscle mass or not. The DSST may be used to predict older mortality in the United States, with results showing greater significance for women and less educated individuals [12]. People with a low DSST have an increased risk of death and disability, even among healthy older persons [11]. Additionally, a higher DSST is linked to a lower chance of passing away in patients undergoing carotid endarterectomy and older persons with high white matter signaling [35, 36]. In our study, DSST was more substantially linked with all-cause mortality in older persons with low muscle mass in both ALM: BMI-defined and ASMI-defined low muscle mass, increasing death by 2.8% and 4.3%, respectively.

This study has a lot of advantages. We examined the relationships between low muscle mass under several definitions and cognitive function and prognosis using three objective criteria to define low muscle mass individually. The co-predictive effect of low muscle mass and DSST on prognosis was examined in this study, adding to the body of research showing that older persons with low muscle mass had worse prognoses when their DSST scores were lower. A thorough evaluation of physical function in older persons with cognitive impairment is therefore important. We ruled out the possibility that variation in individual characteristics such as race, physical activity and lifestyle factors might have influenced our findings. Some limitations in this study must be highlighted. First, the cross-sectional study could not establish a causal relationship between low muscle mass and cognitive impairment. Second, at this age level, NHANES does not collect data on muscle function; nonetheless, cognitive decline and muscle function decrease may be more closely associated, and muscle function may be a stronger indicator of long-term consequences [33]. Third, even though we took into account a number of possible confounders of the outcome, we left out several factors that may have affected the relationship between low muscle mass and cognitive impairment and prognosis, such as the severity of various diseases and information on medication use. Fourth, we counted cardiovascular and cerebrovascular deaths; however, after adjusting for covariates, we did not draw uniform conclusions, most likely due to the small number of cardiovascular and cerebrovascular deaths. We look forward to larger studies of cardiovascular and cerebrovascular death cohorts in the future, which will further clarify the correlation of LMM and DSST with cardiovascular and cerebrovascular deaths.

## Conclusions

Low muscle mass and cognitive function impairment are significantly correlated in the older population. Additionally, low muscle mass and low DSST score, alone or in combination, could be risk factors for mortality in older people. To enhance prognosis, more focus must be given to older persons who also have low muscle mass and cognitive impairment.

### Supplementary Information

Supplementary Material 1.

## Data Availability

The datasets generated and analyzed in the current study are available at NHANES website: https://www.cdc.gov/nchs/nhanes/index.htm.
