# Supplementary material for: Association of low muscle mass with cognitive function and mortality in USA seniors: results from NHANES 1999–2002
Source: BMC Geriatr. 2024 May 11;24:420. doi: 10.1186/s12877-024-05035-9 (PMC11088051; doi:10.1186/s12877-024-05035-9)
Supplement: Supplementary file 1 — Supplementary Material 1. [file 12877_2024_5035_MOESM1_ESM.docx]

Table S1. Cognitive scores in relation to age in different muscle mass subgroups

|  | Age Group | | | |
| --- | --- | --- | --- | --- |
|  | 60–69 years | 70–79 years | 80+ years | p-value^#^ |
| ALM |  |  |  |  |
| LMM | 51.35±19.66 | 42.22±18.09 | 37.60±14.62 | <0.001 |
| Non-LMM | 52.40±17.11 | 44.32±15.82 | 35.05±16.39 | <0.001 |
| P-value* | 0.053 | 0.080 | 0.566 | ---- |
| ALM:BMI | |  |  |  |
| LMM | 45.65±17.21 | 39.14±18.48 | 32.40±14.42 | <0.001 |
| Non-LMM | 52.85±17.34 | 44.66±15.83 | 36.71±15.94 | <0.001 |
| P-value* | <0.001 | <0.001 | 0.009 | ---- |
| ASMI |  |  |  |  |
| LMM | 51.69±20.37 | 38.31±15.70 | 35.43±14.23 | <0.001 |
| Non-LMM | 52.32±17.43 | 44.47±16.29 | 36.16±16.14 | <0.001 |
| P-value* | 0.974 | 0.016 | 0.193 | ---- |

All values are adjusted mean ± SD. Reference category is non-LMM, based on ALM, ALM:BMI or ASMI defined LMM.

Data are weighted according to the National Health and Nutrition Examination Survey protocol

Table S2. Association of low muscle mass (LMM) with all-cause, cardiovascular and cerebrovascular mortality

| OR (95%Cl) P-value | | | |
| --- | --- | --- | --- |
|  | Model 1 | Model 2 | Model 3 |
| Overall Death |  |  |  |
| ALM LMM | 1.853 (1.850-1.856) <0.001 | 1.499 (1.495-1.502) <0.001 | 1.460 (1.456-1.463) <0.001 |
| ALM:BMI LMM | 1.819 (1.815-1.823) <0.001 | 1.614 (1.609-1.619) <0.001 | 1.452 (1.448-1.457) <0.001 |
| ASMI LMM | 2.870 (2.862-2.878) <0.001 | 2.743 (2.733-2.753) <0.001 | 3.075 (3.063-3.088) <0.001 |
| Cardiovascular Death |  |  |  |
| ALM LMM | 0.710 (0.709-0.712) <0.001 | 0.736 (0.734-0.737) <0.001 | 0.782 (0.780-0.784) <0.001 |
| ALM:BMI LMM | 1.101 (1.098-1.104) <0.001 | 1.109 (1.105-1.112) <0.001 | 0.985 (0.982-0.988) <0.001 |
| ASMI LMM | 0.887 (0.884-0.889) <0.001 | 0.866 (0.864-0.869) <0.001 | 0.978 (0.975-0.981) <0.001 |
| Cerebrovascular Death |  |  |  |
| ALM LMM | 1.139 (1.134-1.144) <0.001 | 1.103 (1.098-1.109) <0.001 | 1.210 (1.204-1.215) <0.001 |
| ALM:BMI LMM | 1.203 (1.197-1.210) <0.001 | 1.140 (1.134-1.146) <0.001 | 1.126 (1.120-1.132) <0.001 |
| ASMI LMM | 0.435 (0.432-0.439) <0.001 | 0.389 (0.386-0.392) <0.001 | 0.414 (0.411-0.418) <0.001 |

All values represented are hazard ratios [95% confidence interval]. Reference category is non-LMM, based on ALM, ALM:BMI or ASMI defined LMM.

Data are weighted according to the National Health and Nutrition Examination Survey protocol

Model 1: no adjustment
Model 2: adjusted for age, gender, race, education, annual household income, smoking status, alcohol intake
Model 3: adjusted for model 2 plus hypertension, diabetes mellitus, congestive heart failure, non-skin cancer, stroke, Osteoporosis, arthritis, physical activity
